# Thermographic analysis of perforations in polyurethane blocks performed with experimental conical drill bit in comparison to conventional orthopedic drill bit: a preliminary study

**DOI:** 10.1186/s13104-024-06862-0

**Published:** 2024-07-17

**Authors:** Inácio Bernhardt Rovaris, Anderson Luiz de Carvalho, Gabriel Aardewijn Silva, Daniel Guimarães Gerardi, Marcelo Meller Alievi

**Affiliations:** 1 Department of Animal Medicine, Federal University of Rio Grande do Sul, Porto Alegre, RS Brazil; 2 Department of Veterinary Sciences, Federal University of Paraná, Palotina, PR Brazil; 3 Federal University of Rio Grande do Sul, Porto Alegre, RS Brazil

**Keywords:** Bone drilling, Drill bit design, Infrared thermography, Temperature, Orthopedic drill

## Abstract

**Objective:**

Conical orthopedic drill bits may have the potential to improve the stabilization of orthopedic screws. During perforations, heat energy is released, and elevated temperatures could be related to thermal osteonecrosis. This study was designed to evaluate the thermal behavior of an experimental conical drill bit, when compared to the conventional cylindrical drill, using polyurethane blocks perforations.

**Results:**

The sample was divided into two groups, according to the method of drilling, including 25 polyurethane blocks in each: In Group 1, perforations were performed with a conventional orthopedic cylindrical drill; while in Group 2, an experimental conical drill was used. No statistically significant difference was observed in relation to the maximum temperature (MT) during the entire drilling in the groups, however the perforation time (PT) was slightly longer in Group 2. Each drill bit perforated five times and number of perforations was not correlated with a temperature increase, when evaluated universally or isolated by groups. The PT had no correlation with an increase in temperature when evaluating the perforations universally (*n* = 50) and in Group 1 alone; however, Group 2 showed an inversely proportional correlation for these variables, indicating that, for the conical drill bit, drillings with longer PT had lower MT.

## Introduction

Bone perforation with orthopedic drill bits is present in most orthopedic, orthodontic and neurosurgery procedures [1–3]. During the drill rotations in the creation of bone holes for the introduction of implants, friction is generated at the interface of the drill and bone, releasing energy in the form of heat [4]. Several factors influence the temperature of bone drilling, including tool design, cutting depth, rotational speed, axial loading, irrigation technique, and bone density [4, 5]. Shu et al. evaluated the cellular damage of osteoblasts in the face of elevated temperatures and observed that increasing temperature and its exposure time is directly related to cell death [5]. This type of injury is denominated thermal necrosis.

Thermal necrosis may be related to cell apoptosis and reabsorption, infection, and early loosening of implants, resulting in direct or indirect loss of stability of the osteosynthesis fixation systems [6–8].

Infrared cameras have been used in several studies for conducting thermal tests on bone drilling [4–6, 9–13].

The development of conical orthopedic devices is related to the need to increase implant resistance and provide greater stability for fixation, reducing complication rates [14–16]. The aim of this work is to evaluate the thermal behavior of an experimental conical drill, which presents a possible important mechanical potential, when compared to the conventional orthopedic cylindrical drill. Our hypothesis is that the experimental conical drill does not produce more heat compared to the conventional orthopedic cylindrical drill, when drilling in polyurethane blocks, which would enable it, from a thermal perspective, to be evaluated in future biomechanics assays.

## Materials and methods

### Polyurethane blocks and groups

**Fig. 1:**
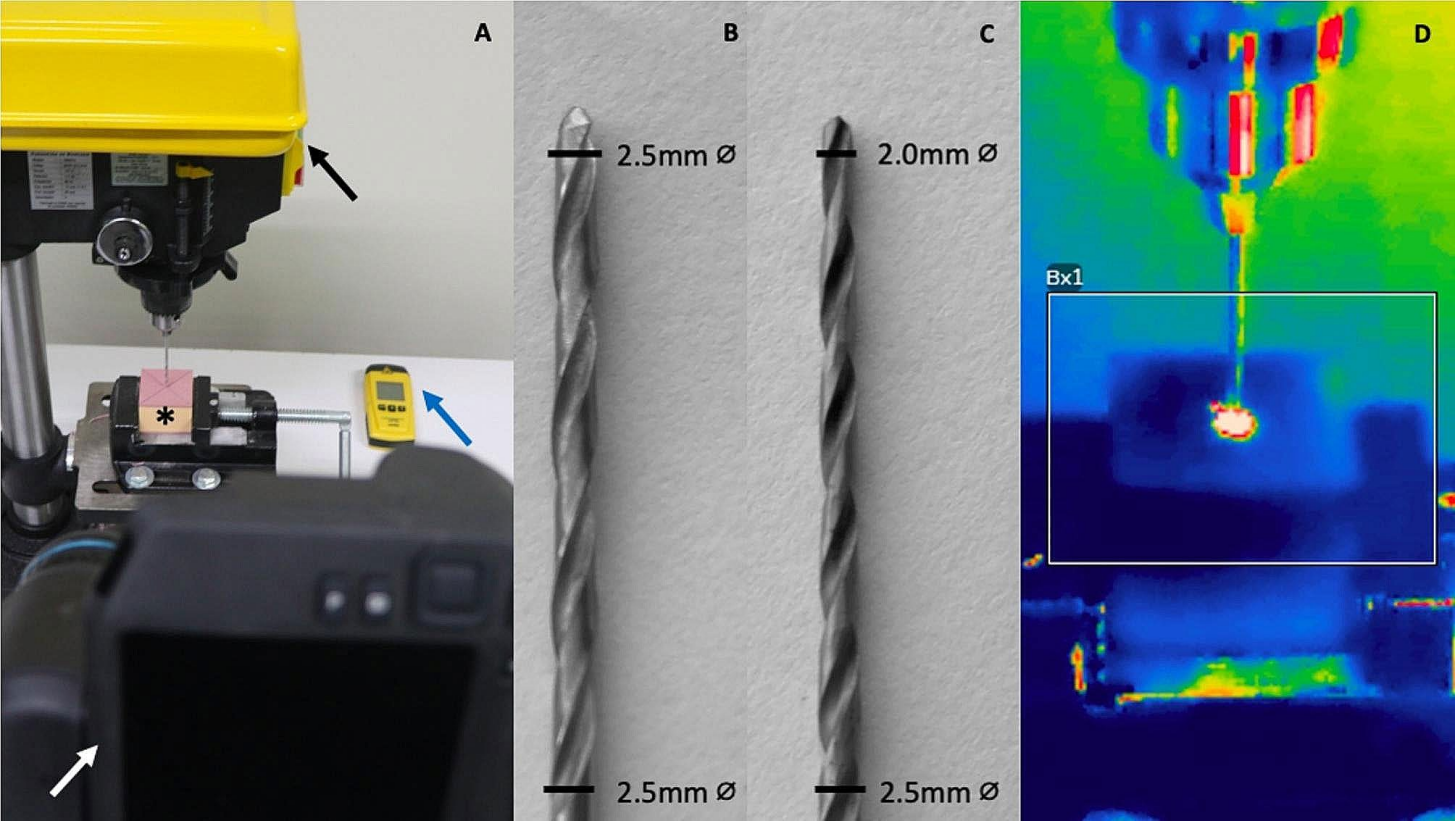
**A**: Studio with opaque colored background for thermographic tests, containing a vertical drilling machine (VONDER^®^ FBV013 1/2 In. 1/3HP) (black arrow), a polyurethane block in a 6-inch bench vise (VONDER^®^) (asterisk), a digital tachometer (VONDER^®^ TDV 100) (blue arrow), and an infrared thermography camera (FLIR^®^ T530, Danderyd, Sweden) (white arrow). **B**: Conventional orthopedic cylindrical drill **C**: Experimental conical drill. **D**: Thermographic image of the perforation

Bicortical polyurethane blocks (PB) (Nacional Ossos^®^, São Paulo, Brazil) were used, with a density of 40 pounds per cubic foot (PCF)/(0.96 g/cm^3^) in the two cortical (2 mm thickness); and 20 PCF/(0.16 g/cm^3^) in its central part (30 mm thickness), which represents the medullary portion. The PB had total dimensions of 47.5 mm x 45 mm x 34 mm (Fig. 1A), respecting the Brazilian Technical Standards Association (ABNT) NBR15678 and NBR15675-4 regulations [17, 18], referring to the use of rigid polyurethane foam for testing implants and the test method for determination of axial pullout resistance, respectively.

For this study, the sample was divided into two groups according to the method of drilling, including 25 PB in each group:

- Group 1 (G1): Drilling with conventional orthopedic cylindrical drill (CCD), 2.5 mm (Fig. 1B).
- Group 2 (G2): Drilling with experimental conical drill (ECD), 2.5–2.0 mm (Fig. 1C).

### Thermographic assessment

Thermographic tests were performed in a studio set up with opaque colored backgrounds and thermally regulated by a temperature conditioning system. It was established that the ambient temperature would be 22 °C and that all equipment, including implants and PBs, would need to be exposed to this temperature for at least four hours, aiming uniformity and thermal stability.

Thermographic images were obtained at 30 fps using an infrared thermography camera T530 (FLIR^®^, Danderyd, Sweden) (Fig. 1A), positioned on a level tripod at a distance of 0.5 m from the PB, with an inclination of 30°, and adjusted to an emissivity of 0.98 for filming. After accommodating and leveling the PB in the vertical drilling machine, a brief thermal stabilization of the PB surface was awaited before drilling was executed, recording the act of drilling and the post-drilling observation period of 40s (Fig. 1D). The images were analyzed by Thermal Studio software v2.0.6 (FLIR^®^, Danderyd, Sweden), considering perforation time (PT) and maximum temperature during the entire drilling (MT) at the perforation area (rectangle highlighted in Fig. 1D).

### Drill bits

In G1, five identical cylindrical drill bits (CCD) made of stainless steel (AISI 420B) (Cãomedica^®^, São Paulo, Brazil) were used, with the same diameter (2.5 mm) and length (150 mm). The length of the shaft was 100 mm, the length of the helix was 50 mm with an angle of 25° and the point angle was 90°.

In G2, five identical experimental conical drills (ECD) were used, made from the same stainless steel (AISI 420B). We also intend to create the same shank profile (100 mm) and helix (50 mm) as the drills used in G1, but due to its conical shape, it presented a progressive reduction in diameter. The helix starts at 2.5 mm, which remains for 15 mm and after that the diameter of the drill begins to progressively decrease until reaching 2.0 mm at its point.

Five drill bits were used in each group (G1 and G2); each drilling five PBs, and each drilling was evaluated separately.

### Polyurethane blocks perforations

To perforate the PBs, a vertical drilling machine was used (VONDER^®^ FBV013 1/2 In. 1/3HP, Paraná, Brazil) with rpm regulation, installed on a level surface and fixed to the ground. To stabilize the PBs during perforation, a 6-inch bench vise (VONDER^®^, Paraná, Brazil) was attached (Fig. 1A).

It was established a rotational speed of 1130 rpm, which was checked before each perforation, using a digital tachometer TDV100 (VONDER^®^, Paraná, Brazil) (Fig. 1A). The depth of each drilling was standardized based on the length of the drill bit, with the purpose of the end of the drill bit crossing 2 mm into the far cortical of the PB. This measurement was performed using a castroviejo specimeter.

The perforations were executed manually and all by the same operator, occurring in groups of ten PBs (five from each group), aiming to maintain the same pattern in all groups, but allowing small individual variations between perforations, as observed in routine surgical procedures. Perforation times (times between the first contact of the drill with the PB until its complete exit) were obtained and MTs were analyzed using Thermal Studio software v2.0.6 (FLIR^®^, Estocolmo, Sweden).

### Statistical analysis

Data were tabulated in Microsoft Excel software v.2016 (Microsoft Corp., Washington, USA). The statistical analysis was performed applying a software program (SPSS Statistics v24.0, IBM Inc. Company, New York, USA). The Kolmogorov-Smirnov test was used to evaluate data for normal distribution. Mean and standard deviation (SD) were used to describe quantitative variables and those were compared between the two groups using the Student’s t-test (MT and PT). Pearsons’s test was used to access correlation between quantitative variables (MT and PT, MT and number of perforations). A *P*-value ≤ 0.05 was considered significant for all analysis.

## Results and discussion

This study evaluated the thermal behavior of perforations in PBs by comparing the use of two orthopedic drill bits with different structural characteristics. Screws inserted in holes drilled by conical drills may have important mechanical potential, and the thermal study of perforations with conical drills is necessary to evaluate the feasibility of their application in future surgical procedures.

**Fig. 2:**
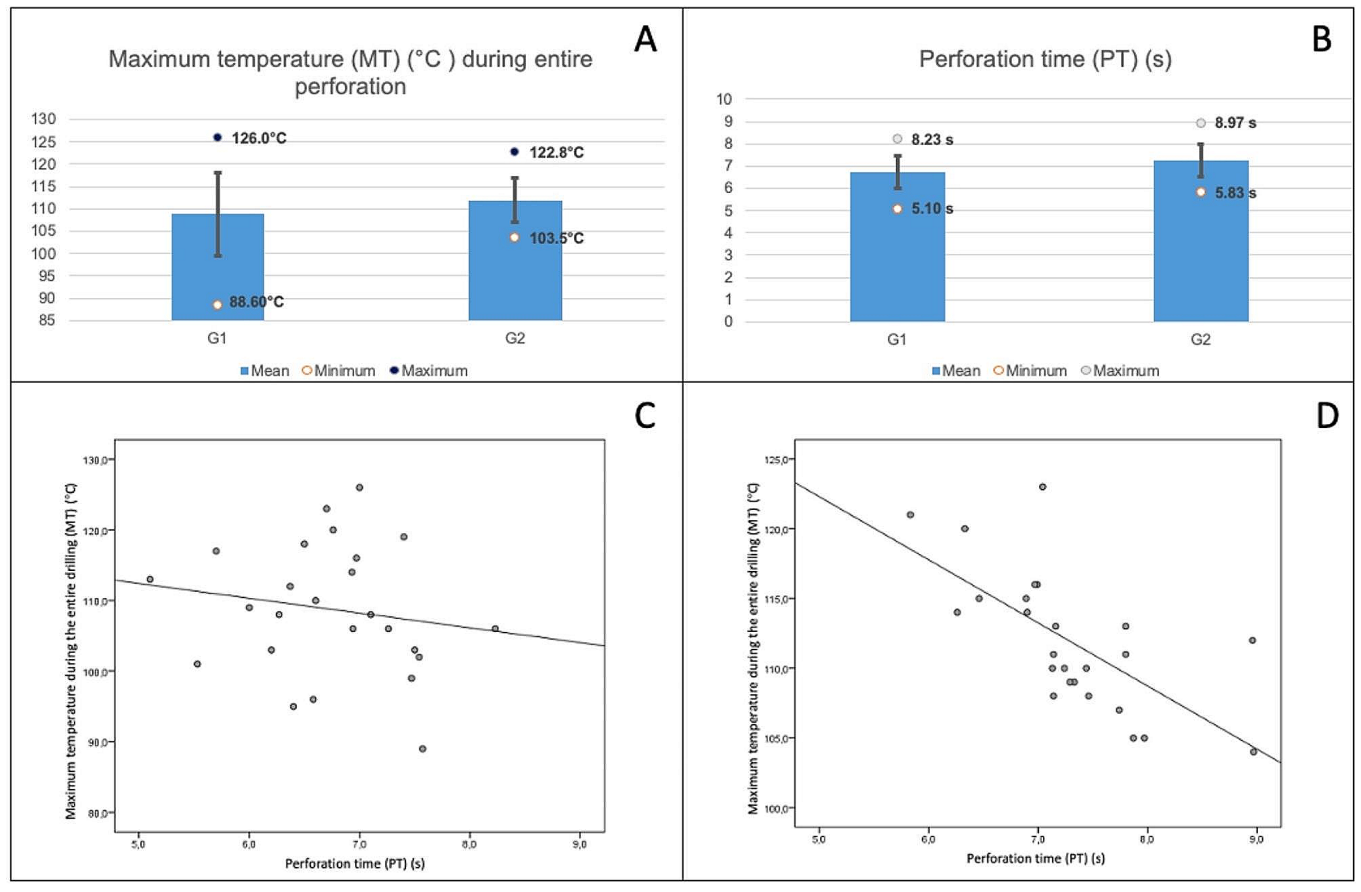
Mean, minimum and maximum values of maximum temperature during the entire drilling (MT) (**A**) and perforation time (PT) (**B**), according to group. The standard deviation is represented by the vertical black bar **(A**,** B**). Correlations between MT and PT in Groups 1 (conventional orthopedic cylindrical drill) (**C**) and 2 (experimental conical drill) (**D**)

In the current work, no statistical difference was observed in relation to the mean MT captured during drillings in G1 and G2 (Table 1). However, G1 presented the highest MT, comparing to G2 (Fig. 2A). Recently, Gehrke et al. evaluated the thermal and histological repercussions of using a conical versus a cylindrical drill bit in drilling rabbit tibias and observed that conical drills generated approximately 10% less heat, a statistically significant difference. In the histological comparison, a larger area of new bone formation was observed after 30 days of drilling, better results than those seen in holes made with cylindrical drills [19]. Shu et al. evaluated in vitro the cellular repercussions of thermal exposure at different temperatures and times for four days, and observed that osteoblasts not only suffered immediate injuries, but also presented important consequences that affected cell viability throughout the follow-up period [5]. In the present study, all MTs from the perforations of both groups were obtained at the drill exit surface, which can be explained by the accumulation of heat due to friction during drilling [5].

**Table 1:** Comparison of the mean maximum temperature during the entire drilling (MT) and perforation time (PT) time between groups 1 (conventional orthopedic cylindrical drill) and 2 (experimental conical drill)

	Group 1 (conventional orthopedic cylindrical drill) (*n* = 25)	Group 2 (experimental conical drill) (*n* = 25)	*P*-value*
Mean maximum temperature during the entire drilling (MT) (°C)	108.82 (± 9.22)	111.98 (± 4.94)	0.14
Mean perforation time (PT) (s)	6.74 (± 0.72)	7.28 (± 0.73)	0.011

*Student’s t-test for independent variables

In the present study, it was observed that there was a statistical difference between the means of the PT of G1 and G2 (Table 1; Fig. 2B). Group 1 had a lower mean PT when compared to G2. However, it is noteworthy that the difference in the mean PT between G1 and G2 was only 0.54s. This time is relatively short when compared to drilling times described by other authors, who evaluated exposure to high temperatures and thermal bone damage from drillings that lasted 15 to 60s [4, 5, 20].

The drill progression speed and, consequently, the PT can influence the drilling temperature. Faster drilling has a shorter heat transfer time to the drilled object. On the other hand, to drill faster, a greater axial force must be applied during drilling, which increases friction, which can lead to an increase in drilling temperature [6, 12]. When the PT and MT of the 50 PBs were evaluated together (G1 + G2), no correlation was observed between these variables (*r*=-0.229; *p* = 0.11). The same was observed when only G1 perforations were evaluated separately (*r*=-0.166; *p* = 0.42) (Fig. 2C), which means, both faster and slower perforations did not influence the MT. Nevertheless, when G2 was evaluated separately, an inversely proportional correlation was observed between PT and MT (*r*=-0.687; *p* < 0.001) (Fig. 2D), meaning longer drillings presented lower temperatures compared to faster drillings. Based on these findings, it is suggested that, for ECD, longer drilling times are preferable, as they produce a lower drilling temperature.

Shakouri and Nezhad evaluated the CCD drilling temperature in bovine femurs, with different drilling times and different rotational speed, and concluded that faster drillings with higher rpm tend to heat up less, for two main reasons: lower contact time of the drill with the drilled object, and greater capacity to eliminate heat through bone chips [4]. However, in the present study it was not possible to observe these thermal behaviors during drilling, neither with CCD nor with ECD.

Each drill bit from G1 and G2 perforated five PBs. No correlation was found between the number of perforations and MT, even when evaluating the groups together (*r* = 0.047; *p* = 0.747) or separately (G1: *r* = 0.014; *p* = 0.948 and G2: *r* = 0.118; *p* = 0.576), suggesting that there was no significant wear on the drill bits to influence the perforation temperature over the five perforations of each drill. Alam et al. related the use of worn drills to the need to increase axial force and drilling time, which caused higher temperatures during bone drilling [8]. However, in the aforementioned work, tests were executed with drills that drilled 50, 100, 150 and 200 times. Therefore, it is feasible to compare the five perforations of each drill in the present study, with no influence on thermal variables depending on the number of perforations. Furthermore, in routine orthopedic procedures, the same drill bit is used to perform several drillings [8], which allows simulating a surgical reality, with respect to the number of perforations, in the present study.

Several studies have evaluated the thermal performance of orthopedic drills and thermal cameras are present in most of these studies, as a non-destructive tool that does not compromise the structure of the drilled component and presents good results [4, 5, 9, 13, 21]. On the other hand, thermocouples, which are also equipment used to evaluate drilling temperatures, need to be installed inside the PBs so that it is possible to measure thermal changes through their sensors [8, 19, 22]. Changes to the PB, such as perforations to install thermocouples, can cause areas of structural weakness, preventing other tests, such as biomechanics, to be carried out with the same component [17, 18]. In this study, we chose to use the T530 camera (FLIR^®^, Danderyd, Sweden), which applies infrared technology to capture images. Shakouri and Nezhad evaluated the two measurement methodologies in drilling bovine bones and the thermal camera presented reliable results, similar to those of thermocouples [4].

In conclusion, there was no difference between the means of the maximum temperature during the entire drilling between the two groups (conventional orthopedic cylindrical drill and experimental conical drill), highlighting that, in polyurethane blocks, the experimental conical drill presents similar thermal behavior compared to the conventional orthopedic cylindrical drill. This result encourages biomechanical tests to be conducted with the experimental conical drill. It was also possible to state that longer drilling times with the experimental conical drill resulted in lower drilling temperatures in polyurethane blocks.

### Limitations

Even though no difference was observed in the mean MT between G1 and G2, and despite ensuring repeatability and homogeneity of PBs, the polyurethane does not have the same thermal characteristics as natural bones [23], and this highlights the need for further studies to endorse the thermal and mechanical performance of conical drills in surgical procedures. Furthermore, cooling techniques such as irrigation were not used during drilling, due to the characteristics of the polyurethane blocks and the lack of knowledge of their behavior in the face of irrigation. Studies with natural bones and largest samples are necessary to enable such an assessment. Another limiting factor for this type of resource is the possibility of irrigation interfering with the capture of images by the infrared camera. Although most studies use natural bones as a model for evaluating the thermal behavior of orthopedic drills, studies such as those conducted by Pazarcı and Gündoğdu [13], Fernandes et al. [24] and Teixeira et al. [25] used synthetic bones for this type of evaluation.

The statistical difference between the PT of G1 and G2 may have influenced MT, however there was a subtle increase in PT of G2 compared to G1. This can be explained by the fact that the drillings were performed manually. However, this difference was less than one second, being smaller than the standard deviation of both variables, which clinically may not be relevant.

## Data Availability

The datasets used and/or analyzed during the current study are available from the corresponding author on reasonable request.

## Abbreviations

CCD: Conventional orthopedic cylindrical drill
ECD: Experimental conical drill
G1: Group 1
G2: Group 2
MT: Maximum temperature during the entire drilling
PB: Polyurethane block
PT: Perforation time
